# Problems lowering the study quality in traditional medicine, introspection from an example of meta-analysis of acupuncture

**DOI:** 10.1186/s12906-019-2806-z

**Published:** 2020-02-11

**Authors:** Qiliang Chen, Qiong Wang, Shanshan Ding, Shunan Li, Yuanyuan Zhang, Shujiao Chen, Xuejuan Lin, Candong Li, Tetsuya Asakawa

**Affiliations:** 1 Research Base of Traditional Chinese Medicine Syndrome, Fujian University of Traditional Chinese Medicine, No.1 Qiuyang Road, Shangjie town, Minhou District, Fuzhou, 350122 China; 2 Hangzhou Changgentang Clinic of TCM, Hangzhou, 310009 China; 3 Department of Neurosurgery, Hamamatsu University School of Medicine, Handayama, 1-20-1, Higashi-ku, Hamamatsu-city, Shizuoka 431-3192 Japan

**Keywords:** Study quality, Traditional medicine, Evidence-based medicine, Meta-analysis, Acupuncture, Intracerebral hemorrhage

## Abstract

**Background:**

Most randomized controlled trials (RCTs) of traditional medicine (such as traditional Chinese medicine (TCM), psychotherapy or behavioral therapy, and dietary interventions, etc.) have reported that they could not provide convincing evidence to support the efficacy because of the low quality of their studies. Here, we aimed to determine the underlying problems of the study quality using standards of evidence-based medicine (EBM) to evaluate the efficacy of traditional medicine.

**Methods:**

We conducted an example of meta-analysis to evaluate the efficacy of acupuncture, a classical treatment of TCM, for treatment of intracerebral hemorrhage (ICH). The quality of the included studies was evaluated by using a Jadad score.

**Results:**

A total of 24 Chinese RCTs that enrolled 1815 patients with ICH were included. Although the results suggested that acupuncture had good efficacy for relief of neurological deficits and improvement of the activities of daily living despite the high heterogeneity of the included studies, the low quality of the included literature reduced the worthiness of the evidence. Two systematic problems (lack of blinding and allocation concealment and high heterogeneity) and one non-systematic problem (lack of reports on adverse events and follow-up) of the TCM studies were found in this illustrational meta-analysis. We believed that other interventions of traditional medicine also suffer from these problems.

**Conclusions:**

Non-systematic problems can be improved by perfecting the experimental design, educating the researcher, and improving the reporting system. However, systematic problems are derived from the characteristics of traditional medicine that are difficult to be corrected. We propose that adoption of objective indexes might be a better solution to improve the systematic problems of traditional medicine. We summarized the problems and the underlying solutions, which may contribute to improve the study quality of systematic review in traditional medicine, strictly complying with the principles of EBM.

## Background

Traditional medicine is defined as a medical system based on the theories, beliefs and experiences indigenous to different cultures involved in the maintenance of health and in the prevention, diagnosis, improvement or treatment of illness. Traditional medicine includes many complex interventions including traditional Chinese medicine (TCM), psychotherapy or behavioral therapy, and dietary interventions, etc.

TCM is an ancient medical system characterized by the concept of wholism (holistic concept) and syndrome differentiation treatment (pattern differentiation) that has been widely practiced in China and Asian areas for thousands of years. Acupuncture, as an important treatment based on the theories of TCM, is accepted as an alternative therapy for treating a broad spectrum of disorders. Since the concept of evidence-based medicine (EBM) was established in the 1990s, it has been accepted by mainstream medicine, but the efficacy of TCM has remained controversial. Many TCM researchers have attempted to provide evidence by conducting randomized controlled trials (RCTs). Unfortunately, most of these so-called RCTs concerning TCM were considered to be weak, flawed, and unable to provide convincing evidence to support the efficacy of TCM if strictly evaluated by EBM standards. In our previous studies on the use of acupuncture to treat neurological diseases, [1, 2] we searched thousands of literature reports regarding the efficacy of acupuncture, but we could not find even one paper that provided convincing evidence. Our recent follow-up study found some RCTs that provided limited evidence for the efficacy of acupuncture in the treatment of Parkinson’s disease, but the evidence was still weak [3]. A recent systematic review by the Cochrane Library regarding Chinese herbs for lithiasis also came to the same conclusion [4]. Thus, almost all reviews or meta-analyses regarding the efficacy of TCM have mentioned the weakness of the included studies and that they cannot draw a certain conclusion regarding efficacy of TCM, and additional well-designed studies are desired in the future. On the other hand, many eminent TCM scientists do not agree that the efficacy of TCM should be evaluated directly by using EBM methods. These scientists think that TCM and Western medicine are derived from different cultures and philosophical foundations [5]. The most important characteristics of TCM are a strong emphasis on dynamic, individual, and wholistic approaches during clinical practice. They believed that is the fundamental reason why most TCM trials were regarded as weak and flawed according to strict EBM standards [5]. Thus, directly using EBM methods might inevitably give a biased result [5–7]. Some TCM scientists want to establish an appropriate novel system to evaluate the efficacy of TCM according to the theory of TCM [8–10].

From a balanced standpoint, it is indispensable to know what happens when we evaluate these TCM studies according to strict EBM standards and to ask if these studies are indeed low quality. Conducting a systematic review or meta-analysis as an example might be the best method to answer these questions because we can summarize and assess numerous literature reports in such a study.

Stroke is the top cause of death and disability in the world, and rehabilitation after stroke is far from satisfactory in China [11]. Most patients undergo TCM treatment (such as acupuncture and massage) instead of modern rehabilitation. Too many Chinese studies have reported that acupuncture delivers good efficacy after stroke. Indeed, acupuncture has an important role in rehabilitation after stroke in China. However, no rigorous meta-analysis has been conducted that strictly followed the guidelines of the Preferred Reporting Items for Systematic Reviews and Meta-Analyses (PRISMA) [12] to evaluate the efficacy of acupuncture on intracerebral hemorrhage (ICH). Additionally, as a follow-up study to our previous studies, [1–3, 11, 13] we conducted an example of meta-analysis on the efficacy of acupuncture for ICH. We attempted to determine the main underlying problems of using an evaluation system based on strict EBM to assess the efficacy of traditional medicine treatment, then provide reasonable solutions. The findings may be useful for improving the quality of study involved in traditional medicine, such as TCM.

## Methods

### Search strategy

We conducted an English-language search of databases, including PubMed, EMBASE, Web of Science, and Google Scholar, by using the terms “acupuncture” OR “electroacupuncture” AND “cerebral hemorrhage” OR “hematencephalon” OR “encephalorrhagia”. We used the same keywords (in Chinese) to search the Chinese database, including Wan Fang Data (http://www.wanfangdata.com.cn/), China Biology Medicine disc (http://www.sinomed.ac.cn/zh/), and China National Knowledge Infrastructure (http://www.cnki.net/). Literatures from firstly available–2018 were included.

Since the aim of this study was investigation of the efficacy of “acupuncture” on “ICH”. The inclusion criteria were RCTs in patients with intracerebral hemorrhagic stroke diagnosed according to the Cerebrovascular Disease Classification (1995) developed by the Fourth National Conference on Cerebrovascular Disease [14] and the updated version. The experimental group must have undergone acupuncture treatment. The exclusion criteria were non-RCTs, case reports, reviews, animal studies, and other types of cerebral hemorrhage, such as subarachnoid hemorrhage. Two independent researchers (QC, YZ) were involved in searching the literature, screening the literature against the inclusion/exclusion criteria by reading the title and abstract to remove excluded study types, and reading the full text to remove studies that did not meet the inclusion criteria. This process was cross-checked and then checked by a senior researcher to ensure the quality and reliability of the included literature (CL). Once the included literature was confirmed, the data, including patient information, treatment, experimental design (sample size, randomization, information of control group, and flaws), and outcome assessment, were independently extracted by the other two researchers (SD, SL). We used the assessments performed in the original text (neurological deficits, overall response rate (ORR) and Barthel Index (BI)), and we did not apply any new assessments. Discussion was performed weekly to resolve any disagreements. All data were finally checked by a third-party author (QW). Consensus was reached for all data before analysis.

### Statistical analysis

This study was conducted strictly according to the PRISMA guidelines [12]. RevMan 5.3 software was used for the meta-analysis. During the homogeneity test, if *p* ≥ 0.1 and *I*^2^ ≤ 50%, the trials were regarded to be homogeneous, and a fixed-effect model was selected, whereas if *p* < 0.1 and *I*^2^ > 50%, the trials were considered to be heterogeneous and a random-effects model was selected. We calculated the standard mean difference (SMD) or weighted mean difference (WMD) for continuous data with a 95% confidence interval (CI).

### Quality evaluation of the included studies

The potential selection bias, performance bias, detection bias, attrition bias, reporting bias, and publication bias were evaluated during the meta-analysis. The quality of the included studies was evaluated according to a standard original Jadad scale described in our previous study [3]. Performing randomization got 1 score. Only those studies performed adequate randomization could be scored as 2 points.

## Results

### Characteristics of the included literature

**Fig. 1:**
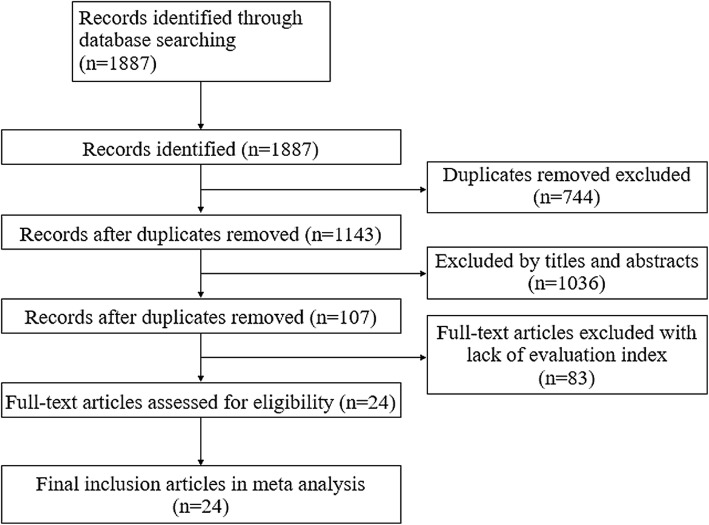
Flow chart of search strategy and selection of the literature reports

A total of 24 RCTs from 1887 studies were included in this study. The characteristics of the included RCTs are listed in Table 1. The included 24 studies were all from the Chinese literature, (no English literature was included, Fig. 1). A total of 1815 patients with ICH were enrolled; of those, 912 were assigned to the acupuncture treatment group, and 903 were assigned to the control group. The age of the patients in five studies were not available, and the average age of the patients in the remaining 19 studies was 59.21 ± 4.42 y in the treatment group, and 59.32 ± 5.23y in the control group. There were no significant differences in the number of patients and age between the treatment and control groups. Regarding the experimental designs, 22 studies used A + C vs. C, and two studies [21, 36] used A + C + R vs. C + R (A: acupuncture, C: conventional therapy, and R: regular rehabilitation treatment by physical therapy, occupational therapy, and speech therapy). The main outcome assessments included the following indexes of neurological deficits: Chinese Stroke Scale (CSS) [6, 15, 17, 18, 20–31, 33–37] and National Institutes of Health Stroke Scale (NIHSS), [16, 19, 32] ORR, and BI. Some studies used indexes, such as calcitonin gene-related peptide (CGRP) [15] activities of daily living (ADL), [22, 24, 30, 36] symptom score of TCM, [38] neuron-specific enolase, [19, 30, 33] hematoma capacity, [27, 38] Glasgow Coma Scale, [19] Fugl–Meyer, [21, 32] S100 [25]; insulin-like growth factor-1 [25]; and interleukin-2 [34] for the outcome measurements (Table 1).

**Table 1:** Characteristics of the included studies in the meta-analysis

Studies	Study design	Age (treatment group)	Age (control group)	Sample size (female)	treatment Intervention	control Intervention	Main outcomes
Bao et al., 2005 [15]	RCT	52.5 ± 11.6	51.4 ± 10.7	60(25)	A + C	C	1.1、2、3、4、5
Cao et al., 2013 [16]	RCT	58.5 ± 14.6	55.5 ± 15.2	60(27)	A + C	C	1.2、2
Chen et al., 2013 [17]	RCT	NA	NA	80 (−)	A + C	C	1.1、2、3
Chen et al., 2016 [18]	RCT	NA	NA	56(28)	A + C	C	1.1
Duan et al., 2010 [19]	RCT	62.20 ± 8.11	60.40 ± 7.85	40(13)	A + C	C	1.2、8、11
Guo et al., 2005 [20]	RCT	54.23 ± 12.73	53.35 ± 11.73	60(23)	A + C	C	1.1、2
Hou et al., 2018 [21]	RCT	61.25 ± 7.56	60.47 ± 8.41	126(47)	A + C + R	C + R	1.1、3、12
Jiang et al., 2010 [22]	RCT	NA	NA	60 (−)	A + C	C	1.1、2、6
Li et al., 2006 [23]	RCT	59.16 ± 12.89	62.82 ± 10.53	100(40)	A + C	C	1.1、2
Li et al., 2008 [24]	RCT	56 ± 8	56 ± 6	96(40)	A + C	C	1.1、2、6、12
Li et al., 2009 [25]	RCT	48.5 ± 5.2	45.7 ± 6.8	66(31)	A + C	C	1.1、2、3、13、14
Li et al., 2011 [6]	RCT	62 ± 10	64 ± 9	120(54)	A + C	C	1.1、3、7、10
Tao et al., 2014 [26]	RCT	62.7	60.5	54(18)	A + C	C	1.1
Wang et al., 2006 [27]	RCT	59.32 ± 4.17	63.62 ± 5.03	64(25)	A + C	C	1.1、10
Wang et al., 2012 [28]	RCT	60.5	59.5	128(54)	A + C	C	1.1、2
Wu et al., 2001 [29]	RCT	63 ± 10	62 ± 9	46(11)	A + C	C	1.1
Zhang et al., 2005 [30]	RCT	NA	NA	62(27)	A + C + O	C + O	1.1、2、3、6、8
Zhang et al., 2005 [31]	RCT	63 ± 2	62 ± 2	95(30)	A + C	C	1.1、2
Zhang et al., 2017 [32]	RCT	63.2 ± 6.3	63.5 ± 6.1	96(51)	A + C	C	1.2、3、12
Zhao et al., 2006 [33]	RCT	60 ± 10	61 ± 7	60(27)	A + C	C	1.1、8
Zhao et al., 2008 [34]	RCT	58.86 ± 9.37	62.64 ± 7.0	43(17)	A + C	C	1.1、15
Zhou et al., 2003 [35]	RCT	66.8 ± 7.1	67.4 ± 6.5	58(21)	A + C	C	1.1、3
Zhu et al., 2006 [36]	RCT	NA	NA	54(−)	A + C + R	C + R	1.1、2、6
Zou et al., 2009 [37]	RCT	54.26 ± 8.78	55.35 ± 7.85	144(54)	A + C	C	1.1、3

1.Neurological deficit score (1.1.CSS; 1.2.NIHSS); 2.Total effective rate; 3.Barthel; 4.CGRP; 5.ET; 6.ADL; 7.Symptom score of TCM; 8.NSE; 9.NDS; 10.Hematoma capacity; 11.Glasgow Coma Scale; 12.Fugl-Meyer; 13.S100;14.IGF-1; 15.IL-2

A = Acupuncture; C = Conventional therapy; R = Rehabilitation therapy; O = Other treatments; NA = Not available

### Evaluation of the efficacy of acupuncture on ICH

#### Efficacy of acupuncture for neurological deficits

**Fig. 2:**
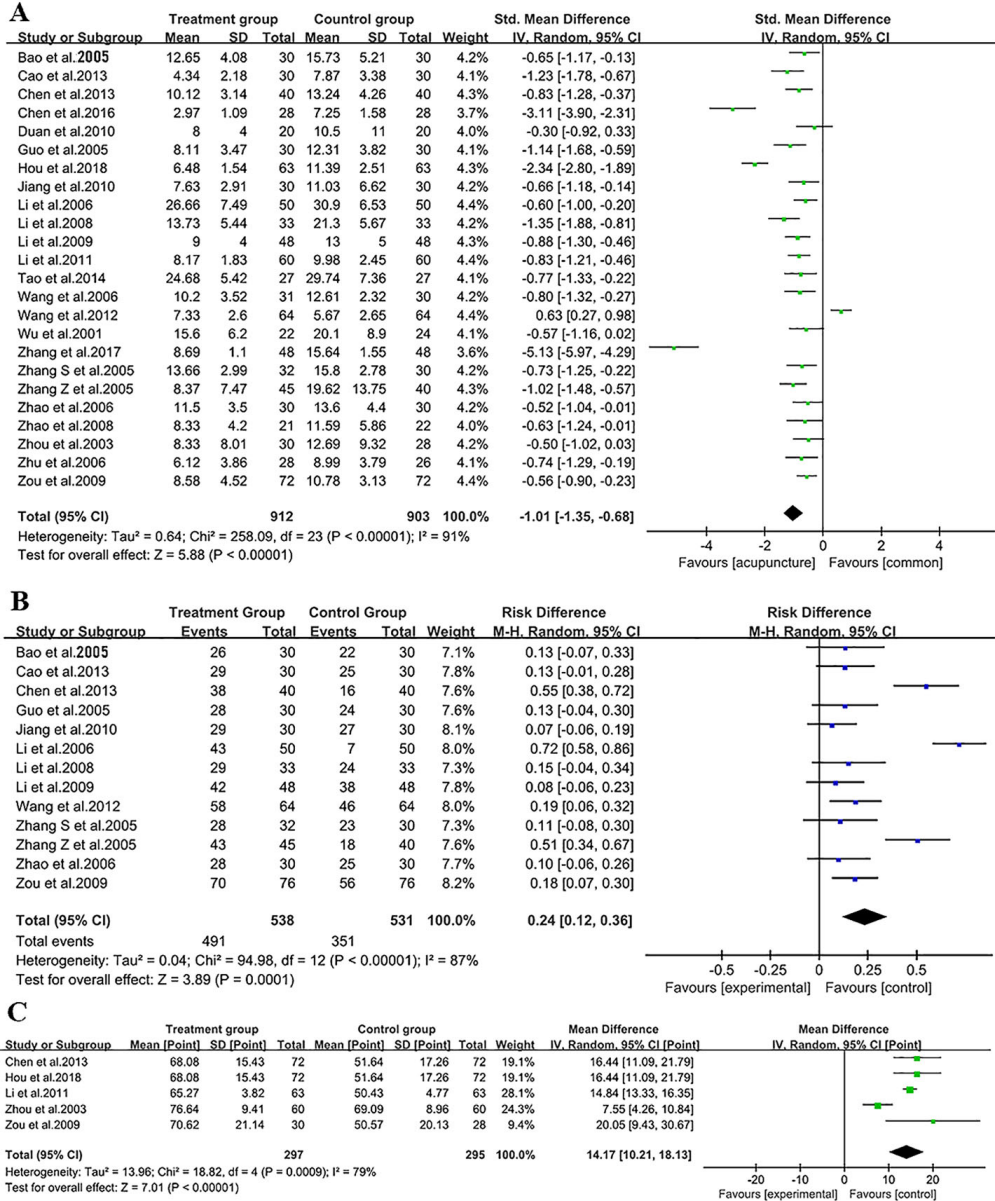
Meta-analysis for the efficacy of acupuncture on ICH. **a** Forest plot for the scores of neurological deficits. **b** Forest plot for the overall response rate (ORR). **c** Forest plot for the Barthel Index

The NIHSS is the most commonly used scale for assessment of neurological deficits in patients with stroke, whereas CSS is a stroke scale widely used in China. CSS is an eight-item clinician-reported scale with a total score of 45 points. For both scales, higher scores represent worse neurological function, and a reduction of the scores indicates amelioration of neurological deficits. As shown in Fig. 2a, all 24 RCTs performed evaluations of the neurological deficits, three studies used NIHSS, [16, 19, 32] and the remaining studies used CSS (Table 1). The homogeneity test (*X*^2^ = 258.09, *p* < 0.00001, *I*^2^ = 91%) indicated extremely high heterogeneity between these studies. A random-effects model was therefore selected to calculate the SMD and 95% CI (WMD = − 1.01, 95%CI [− 1.35 to 0.68, *p* < 0.00001). Despite the high heterogeneity of the included studies, our data suggested that there was a significant reduction in the scores in the acupuncture group. Acupuncture had a therapeutic effect for ameliorating neurological deficits after ICH (A + C > C, or A + C + R > C + R).

#### Acupuncture contributed to enhancement of the ORR to the treatments

A total of 13 studies [15–17, 20, 22–25, 28, 30, 31, 33, 37] evaluated the ORR after undergoing the treatments. However, no study rigorously defined the method used to evaluate the ORR. In the present study, the homogeneity test (*X*^2^ = 94.98, *p* < 0.00001, *I*^2^ = 87%) indicated high heterogeneity among these studies. We selected a random-effects model for analysis. From these data, we concluded that acupuncture significantly improved the ORR (WMD = 0.24, 95%CI [0.12–0.36], *p* = 0.0001) (Fig. 2b).

#### Acupuncture improved the BI score

BI is the most commonly used scale for evaluating ADL after stroke. In this study, five studies [17, 21, 35, 37, 38] evaluated the BI response to acupuncture. The homogeneity test (*X*^2^ = 18.82, *p* = 0.0009, *I*^2^ = 79%) indicated high heterogeneity among these studies. We selected a random-effects model for further analysis. We found that acupuncture significantly improved the BI (MD = 14.17, 95%CI [10.21–18.13], *p* < 0.00001) (Fig. 2c).

### Evaluation of the quality of the included studies and the risk of bias

**Fig. 3:**
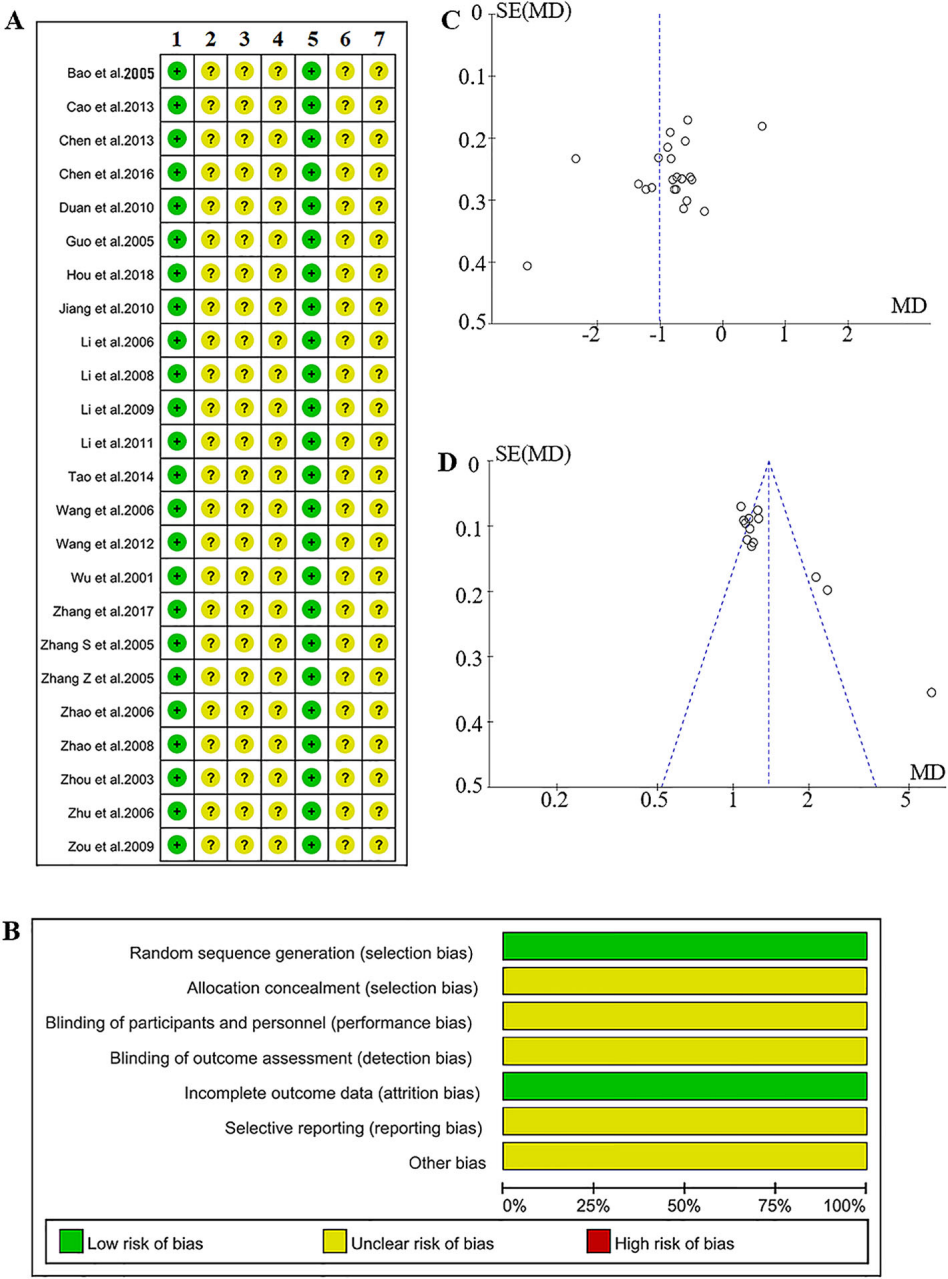
Potential bias risk involved in the present study. **a** Risk of bias of the included literature. 1 = Random sequence generation (selection bias), 2 = Allocation concealment (selection bias), 3 = Blinding of participations and personnel (performance bias), 4 = Blinding of outcome assessment (detection bias), 5 = Incomplete outcome data (attrition bias), 6 = Selective reporting (reporting bias), 7 = Other bias. **b** Summary of the bias risk for the included literature. **c** Funnel plot of the scores of neurological deficits. **d** *Funnel* plot of the overall response rate

The results of evaluating the risk of bias are shown in Fig. 3. All 24 studies were reported as RCTs. Fourteen of the studies introduced their methods for randomization. A random number table was used in eight studies, [16, 17, 20, 23, 26, 28, 31, 34] SAS statistical software was used in four studies, [22, 27, 30, 36] a random number table along with drawing lots was used in one study, [15] and one study selected simple drawing lots [33]. No study mentioned allocation concealment and blinding. Two studies [19, 27] reported withdrawals, and there were no lost cases in the remaining 22 studies. The including studies had a low risk of attribution bias but had a high risk of selection bias (Fig. 3a,b). The publication bias was estimated by using a funnel plot. Both of the neurological deficits (Fig. 3c) and the ORR (Fig. 3d) exhibited an asymmetrical funnel plot, which indicates a potential publication bias. The analyses of the BI could not be evaluated because only five studies were included.

In the evaluation using the Jadad scale, the highest score was in the report by Wang et al., [27] whereas the other studies had BI score of only 1 or 2 points. Most of these studies suffered from two weaknesses: unreported adverse events and no follow-up, and some studies suffered from small samples (Table 2).

**Table 2:** Quality evaluation of included studies

Studies	Jadad Scores	Weaknesses
Bao et al. 2005 [15]	2	NR, NF
Cao et al. 2013 [16]	2	NR, NF
Chen et al. 2013 [17]	2	NR, NF
Chen et al. 2016 [18]	2	SS, NR, NF
Duan et al. 2010 [19]	2	SS
Guo et al. 2005 [20]	2	NR, NF
Hou et al. 2018 [21]	2	NR, NF
Jiang et al. 2010 [22]	2	NR, NF
Li et al. 2006 [23]	1	NR, NF
Li et al. 2008 [24]	1	NR, NF
Li et al. 2009 [25]	2	NR, NF
Li et al. 2011 [6]	1	NR, NF
Tao et al. 2014 [26]	2	SS, NR, NF
Wang et al. 2006 [27]	3	NR
Wang et al. 2012 [28]	1	NR, NF
Wu et al. 2001 [29]	1	SS, NR, NF
Zhang Z et al. 2005 [31]	1	NR, NF
Zhang S et al. 2005 [30]	2	NR, NF
Zhang et al. 2017 [32]	2	NR, NF
Zhao et al. 2008 [34]	2	SS, NR, NF
Zhao et al. 2006 [33]	2	NR, NF
Zhou et al. 2003 [35]	1	NR, NF
Zhu et al. 2006 [36]	2	SS, NR, NF
Zou et al. 2009 [37]	1	NR, NF

SS = Small samples; NR = Adverse events unreported; NF = No follow-up

Although the results of this meta-analysis strongly suggest that acupuncture was effective for treatment of ICH, this conclusion could not be accepted by most of the researchers because of the poor quality of the included RCTs.

## Discussion

In this study, we conducted a standard meta-analysis to assess the problems using the EBM evaluation approach to assess the efficacy of TCM therapy. Although the results of the meta-analysis indicated that acupuncture was effective in treating ICH, the quality of the included studies was quite weak according to the strict evaluation standards of EBM, which limited the worthiness of the evidence. Only limited evidence was obtained from this study, which is in agreement with most of the previous studies involved in TCM evaluation [1–4]. Our results indicated that lack of blinding and allocation concealment and high heterogeneity were the most dominant flaws in most of the TCM trials, which is attributed to the fundamental difference between TCM and Western medicine. In addition, lack of reports of the adverse events and follow-up was also a remarkable problem in most of the TCM studies.

### Summary of the illustrational meta-analysis

In this study, the data of 24 studies showed that acupuncture was effective for relieving neurological deficits after stroke. When using either the NIHSS or CSS, a significant reduction in the scores was observed, which indicated that acupuncture may contribute to amelioration of neurological deficits. ORR also can be significantly improved by acupuncture. Moreover, our data suggest that acupuncture is beneficial for improving the ADL after stroke and enhancement of BI scores. Importantly, two studies in which modern rehabilitation was given [21, 36] concluded that A + C + R > C + R. This result may be inspiring since we know that the modern rehabilitation system is far from satisfactory in China, [11] so appropriate application of acupuncture might compensate for the unmet Chinese modern rehabilitation system. It is noteworthy that the heterogeneity was high in the involved studies. For the aim of this study, we did not perform subgroup analysis and meta-regression analysis. Despite the positive results for efficacy, we obtained negative results concerning the quality of the studies. Although all 24 studies were RCTs with relatively rigorous randomization methods and control group criteria, they suffered from lack of allocation concealment and blinding, and lack of report of the adverse events and follow-up. Consequently, the Jadad scores were low (only one study scored 3, and the others scored 1–2), and there were potential selection, observation, and publication biases. From a strict evaluation standard of EBM, we may conclude that the included studies were poor, and the strength of the evidence gathered from this meta-analysis was low.

Improving experimental designs to provide convincing evidence is a vital problem confronting development of TCM. Yet the awkward situation is that the results of most of the TCM studies strongly suggest that the TCM was effective, but the studies had flawed experimental designs. From the results of the present meta-analysis, we identified two types of problems involved in these studies: systematic problems and non-systematic problems.

There are several limitations in this illustrational meta-analysis: Because the aim of the study did not lie in evaluating the efficacy of acupuncture on ICH; we therefore did not further perform the subgroup analysis to explore the source of heterogeneity. We also did not evaluate the quality of the acupunctural efficacy of the involved studies. These works will be included in our future studies for the aim of evaluating the efficacy of acupuncture on stroke once we can collect enough RCT studies with acceptable study quality. Moreover, this study evaluated many subjective indices like NIHSS, and Barthel, which may also contribute to cause the heterogeneity. Thus, development of novel behavioral assessments for stroke following the principles of OMS (objectification, multipurpose, and simplification) is also proposed [39, 40].

### Systematic problems in the TCM studies

A systematic problem in this study was defined as a problem derived from the characteristics of traditional medicine. Such problems cannot be resolved by simply improving the experimental design because they are caused by the fundamental difference between Western medicine and traditional medicine. Here, we also discuss this problem using the example of TCM. Certainly, EBM originated from modern Western medicine. The essence of EBM is using certain methodology of clinical epidemiology to avoid various biases and attempting to obtain “pure” evidence of efficacy. On the other hand, TCM is from traditional Chinese culture. One of the most important characteristics of TCM is syndrome (TCM syndrome) differentiation treatment (bianzhengshizhi 辨证施治). The therapeutic protocols (such as selection of the acupoints) in the included studies varied because the clinicians had to individually select the best therapeutic parameters according to the TCM syndromes. Sometimes, the same syndrome in Western medicine may be attributed to different TCM syndromes, which require different TCM treatments, and even though in the same patient the TCM syndrome may change in different stages of disease, it will consequently lead to changes in TCM treatment. Thus, TCM treatment emphasizes a patient’s individual requirements, which can be dynamic (the requirements may change after treatment starts and before it ends). The TCM clinicians have to subjectively observe and change the therapeutic regimen momentarily. It is difficult to form a standard therapeutic protocol, as used in Western medicine. On the other hand, most of the current assessments of TCM syndromes are subjective. These are the fundamental difference between TCM and Western medicine. Thus, high heterogeneity, which is caused by the subjective evaluation of the symptoms, and various therapeutic protocols between the trials and included patients, may be the first systematic problem involved in the TCM trials if they are included in a meta-analysis or a systematic review. These problems are also found in the studies evaluating the other traditional medicine.

Another systematic problem is that blinding (especially the double blinding) and allocation concealment is difficult to be practiced during a TCM trial because the clinicians must well grasp the individual and dynamic treatment protocol for each patient. Theoretically, the clinicians can be divided into two groups; one that only provide treatment and another that only assess the outcomes (and are blinded to treatment). However, it is very difficult to realize because most of the current outcome assessments of TCM syndromes are subjective. Different clinician may get different outcome assessment. This is the reason why most of the TCM trials, including the studies involved in the present meta-analysis, suffered from this flaw [2, 4]. However, since the aim of blinding is to avoid observation bias, using objective indexes, of which results cannot be affected by the subjectivity from different observers, may be (the only) solution for this issue. Objectification (for both treatment and assessment) is the tendency and future of TCM. With the development of technologies, such as wearable sensors and mobile internet, many TCM groups are engaging in objectification of TCM syndromes. Li (LC) and his institute are now working on objectification of TCM syndromes, which has been strongly supported by the Chinese government [5, 41].

### Non-systematic problems

We defined a non-systematic problem as a problem that can be resolved by improvement of the experimental design. The most dominant problem is lack of reporting of adverse events and follow-up, which may be because most TCM clinicians and Chinese patients traditionally believe that TCM treatments have *no or few* adverse effects [4]. However, this is not the truth. As early as 2013, our previous study pointed out that acupuncture is not a completely non-invasive treatment [13]. Adverse events of Chinese herbs have also been documented. Ng et al. reported that aristolochic acids were closely associated with onset of hepatocellular carcinomas [42]. A recent study pointed out that abuse of Chinese herbs has been a main cause (26.81%) of drug-induced liver injury in China [43]. Our recent study also reported that some Chinese herbs potentially induce melanosis coli [44]. We believe that these problems also exist in the studies of other traditional medicine interventions. Reinforcing education regarding the adverse events of traditional medicine for both clinicians and patients and improving the reporting systems may be helpful to improve this problem in future trials of traditional medicine [45].

### Other issues regarding the experimental design

Other principles, such as sufficient sample size (desiring a large, multi-center RCT), rigorous experimental design, appropriate statistical analysis, and avoiding confounding bias among other factors are commonly required in a general clinical trial. They are not particular to traditional medicine trials. In the present studies, we found that most of the TCM researchers understood and partly complied with the following principles:

1. Experimental design: All of the studies in this meta-analysis employed T + C vs. C, or T + C + R vs. C + R, which is reasonable. However, in a study involving TCM medicine, a design using T + C vs. C + P (T: therapy of traditional medicine; P: placebo) is recommended [13]. The effects of TCM, especially the efficacy of acupuncture, are often doubted because of the placebo effect. Thus, appropriate development and application of placebo might be important in future TCM study. Placebo acupuncture is also feasible, which has been summarized in our previous study, however, using the placebo acupuncture can only avoid the placebo effect, but cannot fully resolve the problems of “lack of blinding” [13].
2. Randomization: It is very important to randomize patients to avoid selection bias. Most of the included studies reported the use of randomization. It is feasible to apply randomization in a TCM study.

Taken together, according to the principles of EBM, we found that lack of blinding and allocation concealment was the most dominant flaw in traditional medicine trials, and if a meta-analysis is selected for evaluating traditional medicine studies, the high heterogeneity problem should be carefully considered. Objectification of symptoms is the best solution for lack of blinding. The other non-systematic flaws, such as lack of reporting of the adverse events and follow-up, can be satisfactorily improved by perfecting the experimental design, educating the researchers, and improving the reporting system. In this regard, developing objective assessments for symptoms (including TCM syndrome) as well as designing satisfactory placebos (medicine and acupuncture) may be the most urgent task for researchers of traditional medicine. Since blinding and allocation concealment are difficult to be realized in most of the traditional medicine systems, adoption of objective indexes might be a more reasonable solution to improve the systematic problems of trials evaluating traditional medicine, including TCM. We listed the main existing problems along with the related solutions in Table 3. We believe that the study quality of systematic review in traditional medicine will be remarkable improved if these solutions can be seriously noticed and applied (Table 3).

**Table 3:** Summaries of the flaws and solutions for improving the study quality of systematic review in traditional medicine

Problems	Flaws caused by the problems	Solutions
Systematic problems		
Subjective assessments of symptoms	High heterogeneity	Objectification of the assessments of symptoms
Individual treatments.	Difficult to practice blinding and allocation concealment	
Non-systematic problems		
Lack of reporting of adverse events	Lower the reliability of the traditional medicine	Improving the spontaneous reporting system of adverse event; education and training the medical stuff.
Lack of follow -up	Lack of the data of long-term efficacy, lower the evidence of efficacy regarding a therapy of traditional medicine.	Improve the experimental design, adding the contents of follow-up.
Other problems		
Placebo effects	Exaggeration of the efficacy	Adoption of the experimental design using T + C vs. C + P
Small sample size	Lower the reliability of the traditional medicine	Improve the experimental design guided by a statistician.

T: therapy of traditional medicine; C: conventional therapy; P: placebo

## Conclusions

Although the results of this meta-analysis supported the efficacy of acupuncture for ICH, the low quality of the included studies reduced the worthiness of the evidence. The most dominant problems involved in these studies were lack of blinding and allocation concealment and high heterogeneity, which are systematic problems derived from the principles of traditional medicine. Lack of reporting of the adverse events and follow-up was another important non-systematic flaw that can be improved by perfecting the experimental design. Adoption of objective indexes may be a better solution to resolve the systematic problems of traditional medicine. A number of solutions were proposed which may contribute to improve the study quality of systematic review in traditional medicine, strictly complying with the principles of EBM.

## Data Availability

The data and materials used are included in the manuscript.

## Abbreviations

A: Acupuncture
ADL: Activities of daily living
BI: Barthel Index
C: Conventional therapy
CGRP: Calcitonin gene-related peptide
CI: Confidence interval
CSS: Chinese Stroke Scale
EBM: Evidence-based medicine
ICH: Intracerebral hemorrhage
NA: Not available
NF: No follow-up
NIHSS: National Institutes of Health Stroke Scale
NR: Adverse events unreported
ORR: Overall response rate
P: Placebo
PRISMA: Preferred Reporting Items for Systematic Reviews and Meta-Analyses
R: Rehabilitation therapy
RCT: Randomized controlled trial
SMD: Standard mean difference
SS: Small samples
T: Therapy of traditional medicine
TCM: Traditional Chinese medicine
WMD: Weighted mean difference
